# Cooperation in Online Conversations: The Response Times as a Window Into the Cognition of Language Processing

**DOI:** 10.3389/fpsyg.2019.00727

**Published:** 2019-04-09

**Authors:** Baptiste Jacquet, Jean Baratgin, Frank Jamet

**Affiliations:** ^1^P-A-R-I-S Association, Paris, France; ^2^Laboratoire CHArt, Université Paris VIII & EPHE, Paris, France; ^3^Institut Jean Nicod (IJN), École Normale Supérieure (ENS), Paris, France; ^4^Université de Cergy-Pontoise, Cergy-Pontoise, France

**Keywords:** conversational expectations, pragmatics, relevance, Turing test, natural language, cooperation, online conversations

## Abstract

Measuring the cognitive cost of interpreting the meaning of sentences in a conversation is a complex task, but it is also at the core of Sperber and Wilson's Relevance Theory. In cognitive sciences, the delay between a stimulus and its response is often used as an approximation of the cognitive cost. We have noticed that such a tool had not yet been used to measure the cognitive cost of interpreting the meaning of sentences in a free-flowing and interactive conversation. The following experiment tests the ability to discriminate between sentences with a high cognitive cost and sentences with a low cognitive cost using the response time of the participants during an online conversation in a protocol inspired by the Turing Test. We have used violations of Grice's Cooperative Principle to create conditions in which sentences with a high cognitive cost would be produced. We hypothesized that response times are directly correlated to the cognitive cost required to generate implicatures from a statement. Our results are coherent with the literature in the field and shed some new light on the effect of violations on the humanness of a conversational agent. We show that violations of the maxim of Relation had a particularly important impact on response times and the perceived humanness of a conversation partner. Violations of the first maxim of Quantity and the fourth maxim of Manner had a lesser impact, and only on male participants.

## 1. Introduction

The recent advances in Artificial Intelligence (AI) have enabled the spread of virtual social agents in many areas, in particular as customer service agents (Chakrabarti and Luger, 2015; Cui et al., 2017; Xu et al., 2017), but also as coaches providing help to manage psychological issues like depression or anxiety on a daily basis, like Woebot^1^ or Tess^2^. These agents often take the shape of chatterbots (or chatbots): they are agents conversing with a user through a textual conversation using, in general, imitations of natural language comprehension and generation.

While considerable progress has been made in the two fields of syntactic (see Socher et al., 2010; Chen and Manning, 2014, for examples) and semantic processing (see Berant and Liang, 2014; Pasupat and Liang, 2015, for examples), one aspect of natural conversations is often forgotten: pragmatic processing (see Jokinen and De Smedt, 2012; Jacquet et al., 2019, for reviews). Understanding the structure of an utterance and its semantic content is not enough to have a complete understanding of the utterance itself within its context. Indeed, there can be vast differences between what is *said* and what is *meant* in conversations between humans as Grice (1975), and later Sperber and Wilson (1995) noted.

On this distinction between what is *said* and what is *meant*, Grice (1975) introduced the Cooperative Principle along with its maxims to describe various expectations that allow conversation partners to infer the meaning of an utterance through the intention of its speaker. The Relevance Theory (Sperber and Wilson, 1995) later updated Grice's original principles and offered a more in-depth, more unified explanation of the processes involved in inferring what is *meant* from what is *said* (and from what is *not* said).

Because this pragmatic processing requires the virtual agent to have an understanding of the expectations of its user, chatterbots are to this day still struggling with it. The most advanced chatterbots today (Like Cleverbot^3^, and to a greater extent Zo^4^ and those using Watson^5^) are reasonably convincing in their ability to answer questions and maintain terse, simple conversations, but their utterances quickly lose relevance after a couple of sentences, sometimes even as soon as in their second utterance (Especially for Cleverbot). In Zo, which is arguably one of the most convincing conversational agents, this lack of relevance is dodged by the tone of the conversation, as she will often answer with humoristic utterances, including using internet *memes*^6^, that actually let the user find an interpretation of the agent's reply that is relevant to them, despite how generic the answer was.

While this makes for amusing conversational agents that are interesting to play with for a little while, this lack of relevance makes them unusable in more serious environments, where a lack of pragmatic reasoning cannot be hidden behind a shallow but seemingly witty reply. During a conversation with a customer service agent, for example, users will expect the agent to be helpful, not witty (Chakrabarti and Luger, 2015).

Despite the importance of pragmatic processing in how believable a conversational agent is, evaluating its quality within conversations is difficult to automate. Unlike syntactic processing, there are no well-defined rules that are stable enough to base it on, such as grammar. Unlike semantic processing, there are no direct and stable associations between certain speech features and implied meaning.

As a result, there is no gold standard to evaluate the quality of a conversational agent, and even less so to assess the quality of its pragmatic processing, even though many different evaluation methods exist for conversational agents in general (Paroubek et al., 2007; Hung et al., 2009; Ptaszynski et al., 2010; Chakrabarti and Luger, 2015; Meira and Canuto, 2015).

We argue that the Turing Test, already well known in computational sciences as a suggested method to test the (supposed) intelligence of a machine in textual conversations through a comparison with a human, can, in fact, be enough to detect flaws in pragmatic processing during such conversations when it is instead seen as a humanness testing environment. We also argue that the discriminating power of the Turing Test in this context can be made more specific, at the level of the utterance, if it is associated with a recording of the response times (RT) to indicate how difficult inferring the utterance's meaning was.

To test this idea, we used three of Grice's maxims commonly violated in conversations with artificial agents. These maxims are the first maxim of Quantity, the maxim of Relation and the fourth maxim of Manner, which all have been shown to have different effects on the humanness of a chatterbot (Saygin and Cicekli, 2002). We hypothesized that conversational agents violating the maxims would be identified as being more machine-like (Especially for violations of the maxim of Relation) than conversational agents producing more typical utterances.

We also hypothesized that the RT would increase following utterances requiring a high cognitive effort to infer their meaning. In particular, we expected that the responses of the participants would be delayed the most following violations of the maxim of Relation, a lower or absent increase following violations of the maxim of Quantity, and an intermediary increase following violations of the maxim of Manner.

## 2. Theoretical Background

### 2.1. Pragmatics of Conversation

Language is a code encrypting concepts into symbols that are the words of a sentence. However, this code does not contain all the information necessary to retrieve the complexity of the concepts it references. Indeed it only includes the information required to bring the mental representations of the conversational partner which is in the position of receiver slightly closer to those of the conversational partner in the position of the emitter. This operation gives the emitter the opportunity to save processing time (cognitive effort) and to only spend it on encoding information that they believe their partner does not already have. The details that are not important to reach the goal of the conversation can be left out, letting the receiver fill in the gaps with their knowledge, and in doing so avoiding the need to deal with redundant information. This balance between a cost (processing time) and effect (the modification of the receiver's mental states) has been described in the Relevance Theory (Sperber and Wilson, 1995, 2015).

Relevance Theory considers relevance to be the result from the interaction between an utterance's effect on the reader's mental representations, and the processing cost that was required to infer its meaning, using contextual clues along with the utterance itself. The higher the processing cost, the lower the relevance, and the higher the cognitive effect, the higher the relevance of the utterance. As a consequence, optimal relevance is reached whenever an utterance gives a high cognitive effect for a low processing cost. It is also important to consider that, according to this theory, participants in a conversation will by default assume the relevance of an utterance, and that it is worth processing to recover its implied message.

In many cognitive tasks, the behavior of participants could be considered to be biased when compared to formal logic. Relevance Theory, by giving good predictions of these behaviors, instead revealed that people are not incoherent in the way they reason as they use contextual information in addition to the information explicitly provided to them, which makes it difficult to reason without a concrete context. The literature is quite abundant in this area and includes the logic of connectors (see for examples Politzer, 1986; Sperber et al., 1995; Noveck, 2001), Piaget's inclusion task (see for example Masson et al., 2016b; Politzer, 2016), bias in probability judgment (see for examples Hilton, 1995; Baratgin and Noveck, 2000; Baratgin and Politzer, 2006, 2007, 2010), and decision making (see for examples Bless et al., 1998; Bagassi and Macchi, 2006; Masson et al., 2016, 2017a).

Relevance Theory was initially inspired by Grice (1975)'s work, before unifying it and expanding it. When trying to describe what conversations are about, Grice noted that:

“Our talk exchanges do not normally consist of a succession of disconnected remarks, and would not be rational if they did. They are characteristically, to some degree at least, cooperative efforts; and each participant recognizes in them, to some extent, a common purpose or set of purposes, or at least a mutually accepted direction. This purpose or direction may be fixed from the start (e.g., by an initial proposal of a question for discussion), or it may evolve during the exchange…But at each stage, *some* possible conversational moves would be excluded as conversationally unsuitable” (Grice, 1975, p. 45, emphasis in the original).

It is following this description that he proposed the Cooperative Principle, defined as:

“Make your conversational contribution such as is required, at the stage at which it occurs, by the accepted purpose or direction of the talk exchange in which you are engaged” (Grice, 1975, p. 45).

In this principle, Grice explained that participants in a conversation have expectations regarding the shape and content of their partner's utterances. He categorized them into four different maxims and their sub-maxims.

#### 2.1.1. Quality

The first of these maxims is the maxim of Quality: “try to make your contribution one that is true.” It is subdivided into two sub-maxims: (1) “do not say what you believe to be false,” and (2) “do not say that for which you lack evidence.”

Grice considered the maxim of Quality to be the one on which all three others depend, and it can even be argued that the entire Cooperation Principle relies on the receiver of the utterances considering that the emitter believes that what is being said contains a valid information. Otherwise, the receiver would not even try to infer a hidden meaning. In consequence, while this maxim does not depend on the other three, the opposite cannot be said to be true, as all three heavily rely on the maxim of Quality being respected (Benton, 2016).

One can also argue that this maxim might heavily depend on the occidental interpretation of what a lie is, as it uses the term “try [to make your contribution one that is true],” yet in Mopan culture, for example, falsehood is not considered to be depending on the mental state of the emitter and whether they believe that their utterance is true or not: regardless it will be deemed to be a violation of conversational Quality if the information in and on itself can be considered to be false (Danziger, 2010).

It is also worth considering the fact that the sub-maxim of truthfulness (“do not say what you believe to be false”) is often violated (or at the very least suspended) in cases like metaphors and irony, while the maxim of Quality itself is not, since these sentences still do imply a true information despite what is being said being factually wrong. Thus, they cannot be considered to be actual lies, which are violations of the sub-maxim of truthfulness *and* of the maxim of Quality while making the interlocutor believe that they are in fact being respected (Wilson, 1995).

Despite its importance, it cannot be said that its violation produces an actual effect on the humanness of an interlocutor during a conversation (Saygin and Cicekli, 2002). In consequence, this maxim will not be the main focus of our study.

#### 2.1.2. Quantity

Grice's second maxim is the maxim of Quantity, which explains the expectations regarding the actual amount of information contained within an utterance and is defined by two sub-maxims: (1) “Make your contribution as informative as is required (for the current purposes of the exchange),” and (2) “do not make your contribution more informative than is required.”

The first sub-maxim of Quantity is quite often violated, and what its violation implies is usually simple to interpret. For example, it is very commonly used when the emitter of an utterance attempts to deceive the receiver to keep some pieces of information hidden to them (McCornack, 1992). It can also be used to imply that the emitter does not know the answer to a question, and potentially does not care about it either, for example:

1. Where does Marc live?
2. Somewhere on Earth.

In this case, 2 answers with an information that was already well known to 1. As a consequence, this utterance has a low relevance, since it has no cognitive effect, other than potentially changing the perception of the state of interest of 2 in the conversation. Though a violation of the sub-maxim does not necessarily imply a lack of interest. For example:

1. Where does Marc live?
2. Somewhere in France.

In this case, 2 likely does not give enough information (unless the context is specifically to be talking about countries), but this answer might simply be the result of not wishing to violate the maxim of Quality: not giving more information than what they know to be true.

Engelhardt et al. (2006) used an experimental protocol similar to the one used by Tanenhaus et al. (1995) consisting in showing pairs of participants some items to move, and where they can potentially be moved to. One needed to describe which spot each item should be moved to, while the other needed to do the corresponding action of moving the item accordingly. The study showed that participants preferred to over-describe the items to be moved (and in doing so violated the second sub-maxim of Quantity) while trying to avoid under-descriptions, which caused ambiguity. Violations of the maxim in the instruction they received had a direct effect on participants with the task of moving the item: the under-description visibly confused the participants, as was shown through the investigation of their ocular fixations. Confusion has also been observed when over-describing since participants needed more time to understand the movement that needed to be done. Similar results had already been published before by Spivey et al. (2002).

Horn (1984) interpreted the first maxim of Quantity to be the result of the principle of economy. Indeed, the speaker tends to only be giving the information that he or she must (“make your contribution necessary”), and no more. At the same time, they also tend to be giving as much information as possible that facilitates the listener's task by improving the clarity of the meaning of the utterance (“make your contribution sufficient”), and no less. He argues, like Grice hinted at, that the second maxim of Quantity is more related to not giving irrelevant pieces of information, rather than actually giving as little information as possible. This is entirely coherent with the results of the previously described experiment, and more generally with Relevance Theory itself.

In the context of AI in conversations, the over-description is often seen by participants as a somewhat mechanical and artificial behavior, yet the lack of information, which can cause ambiguity and violates the first maxim of Quantity, is actually considered to be more human-like as it can be interpreted to be a sign of disinterest (Saygin and Cicekli, 2002).

According to the Relevance Theory, in the case of the first sub-maxim of Quantity, the emitter of the utterance gives little information, preventing the production of any cognitive effect on the mental representations of the partner. It is possible that they might try to infer another meaning though, which might require a certain cognitive cost, but we expect that it would not be very high, especially since a more likely explanation can be that this person is bored, rude, or not talkative (Saygin and Cicekli, 2002).

In our preliminary experiment, no significant detrimental effect of violations of the first sub-maxim of Quantity could be observed on the humanness of the conversation partner violating it, which was coherent with this observation. In this study, we will continue to be focusing on this sub-maxim as a reference for a low machine-like effect.

#### 2.1.3. Relation

The third maxim of Grice's Cooperative Principle is the maxim of Relation: “be relevant.” Grice explained it with the following example:

“I expect a partner's contribution to be appropriate to immediate needs at each stage of the transaction; if I am mixing ingredients for a cake, I do not expect to be handed a good book, or even an oven cloth (though this might be an appropriate contribution at a later stage)” (Grice, 1975, p. 47).

It is in fact as an attempt to further study this maxim that Sperber and Wilson (1995) suggested the Relevance Theory as a more general explanation of Grice's Cooperative Principle.

Because conversation partners will always assume by default that an utterance is somehow relevant, apparent violations of this maxim have a very noticeable effect. People will indeed believe that the emitter of such utterances is not comfortable with the topic and want to switch to another one. In the context of an artificial partner, this will usually be understood as a lack of comprehension of the sentence the violation was in response to, and as a consequence, it will be qualified to be very machine-like (Saygin and Cicekli, 2002).

In our preliminary experiment, violations of this maxim had a significant effect on the humanness of the emitter of such utterances and on the response times of the utterances following it, especially with female participants. We will continue to use violations of this maxim in our experiment as a reference for a strong machine-like effect.

#### 2.1.4. Manner

Finally, Grice's fourth maxim, the maxim of Manner, is defined through four sub-maxims: (1) “avoid obscurity of expression,” (2) “avoid ambiguity,” (3) “be brief (avoid unnecessary prolixity),” and (4) “be orderly.” The maxim of Manner refers to the structure of the utterance itself and on the inferences resulting from it. Among its sub-maxims we find “be orderly” which relates to the order of the pieces of information given during the utterance. It is not always clear which order should be considered the correct one though. It could very well be placing causes before consequences like in the following example:

1. They took a shower and went out.
2. They went out and took a shower.

In this case, it is clear that 1 and 2 do not have the exact same meaning. Yet the effect of order is not always as visible, as in the following case:

1. It is raining outside; she took her umbrella.
2. She took her umbrella; it is raining outside.

Blackmore and Carston (2005), in their paper on the *and* connector, suggested that keeping a chronological order is not necessarily required in some situations of causality, and that different orders might implicate, among other things, an aspect of surprise, as in their example:

1. Paul can't spell and he is a linguist.
2. Paul is a linguist and he can't spell.

In both cases, the second part following the *and* is the part triggering the effect of surprise, as in 1 Paul is not expected to be a linguist since he cannot spell, and in 2 he is not expected not to be able to spell since he is a linguist. Wilson and Sperber (2012) suggested that the meaning inferred from an utterance with structures similar to these ones is produced from a call to our knowledge on the probabilities of a causality between the different elements of the utterance.

In our experiment, some focus will be given to the fourth sub-maxim of Manner, which was not studied in our preliminary experiment.

### 2.2. Turing Test

Unfortunately, it is quite difficult to study pragmatic aspects of language in an ecological setting. Studies are indeed only qualitative since producing operational protocols for a quantitative study can be quite complex due to the inherent variability of conversations (Cohen, 1971; Alba Juez, 1995; Blackmore and Carston, 2005; Herring, 2013, for examples). We believe that the recent advances in the field of AI offer a new framework in which original methods can be designed to investigate the nature of conversations. Artificial conversational entities are indeed quite common now making them potentially useful tools to study human behavior and, specifically for our study, participants can now readily expect having to interact with them.

The Turing Test (Turing, 1950) is one of such protocols involving an AI. It consists in having participants acting as judges, attempting to find the machine between two interlocutors. Since the task essentially revolves around the participant's ability to compare two interlocutors within an interactive textual conversation, it can be used to explore the conversational features that might be expected when conversing with a human. Because of this, we suggest that involving an actual AI can become superfluous in this context, as participants only need to believe that they will be conversing with one.

The possibility to be talking to an AI today is indeed no longer null because of how widespread they have become in many aspects of our lives, such as personal assistants like Siri^7^, Cortana^8^, Watson^9^, and many others.

Turing (1950) described the idea of a test able to answer the question “Can machines think?” In his paper, he suggested a test analogous to a gender guessing game, where two persons (A and B) of different gender try to convince the participant C that they are the woman while the participant knows that only one woman is present. C engages in a textual conversation with each person, before guessing which of the two character he or she has been talking to is indeed the woman between A and B. Turing suggested replacing A with a machine.

It is worth noting that the Turing Test was initially assumed to be testing the intelligence of machines and on this aspect received many critics. Such critics do not apply to the context of our study since the intelligence of the conversational partner is not what is of interest here. What is essential is the Test's usefulness in assessing the humanness of this conversational partner's behavior. Some unclear aspects of the initial Turing Test still need to be debated though. In particular, whether the participant should or should not be aware of the presence of an AI within the test, as the interpretation of non-human-like behavior could depend on this.

Saygin and Cicekli (2002) indeed showed that participants asked to elaborate on their subjective feelings toward the productions of an AI (without giving them the information that one was in fact present) have a tendency to identify it as an odd behavior, but still human: “Are some of these people mentally ill?” (Saygin and Cicekli, 2002, p. 250). In their experiment, a group of participants was told, in a first phase, to answer a questionnaire on the violation of Grice's Maxims, then was given another questionnaire on the artificial behavior of one of the interlocutors within a conversation. The other group was given the same task in the opposite order. In the first group, the answers to the second questionnaire were much more radical than in the second group, indicating that having an understanding of the maxims helps to determine the humanness of a conversational agent's behavior.

Yet the violation of some maxims seems to have a positive influence on the humanness of an interlocutor for the participants:

“Sometimes maxim violations can create a human-like effect. In fact, strong violations of [Manner] have invariably created favorable impressions. It can be inferred that, had the programs that used being rude or obscure as a strategy been more successfully designed to handle the syntactic components of natural language, they would have appeared quite close to human beings, albeit strange ones. If in addition to this, the semantic processing had included ways to partially handle relevance and quantity, some of these might even have passed the Loebner Test” (Saygin and Cicekli, 2002, p. 254).

If today no artificial agent has managed to pass the Turing Test, some present interesting features. These chatterbots do not necessarily require to be able to learn on their own, especially the oldest of them. They indeed often use keywords in their interlocutor's utterances to generate an answer. The most well-known examples of such programs are ELIZA (Weizenbaum, 1966), A.L.I.C.E. (Wallace, 2009), and more recently Zo.

Zo is as of today a standout among conversation agents. Still, it is far from perfect at keeping track of conversations, like the two previous chatterbots. Indeed, when talking about something like the beach, it might say that it does not like the beach because it does not like sand. When the user replies “Yeah, sand is annoying,” it might reply “Yuo are annoying”^10^. On this specific point A.L.I.C.E. does slightly better in some cases, as for example, if it asks about the number of children, siblings or pets, and if then the user answers “I have two,” the chatterbot will answer “What are their names?” which is an expected question, but will say the same thing if the topic is about computers instead of children (clearly indicating that this is a generic and pre-programmed reply). The lack of pragmatic processing is indeed extremely common in artificial social agents in general (Jacquet et al., 2019) despite experimental data showing its importance, including with social robots (Masson et al., 2017b; Jamet et al., 2018).

Because the chatterbots available are not specific on which maxims they violate, we will avoid using an AI in our experiment in order to clearly dissociate different kinds of violations, and also on account of the potential impact of different elements like vocabulary and grammar. We will still introduce one interlocutor as being an AI, as it is necessary to make participants reflect on what they expect of a human compared to what they expect of an AI.

### 2.3. Response Times

It is common in experimental psychology to record the delay between a stimulus and a response from participants. However, to our knowledge, studies investigating online conversations using them remain very rare (Jacquet et al., 2018).

The concept of using such measures to develop interpretations of the inner workings of the mind is not new. It is based on the general assumption that the human brain is not unlimited in its processing speed as the communication between neurons is not immediate. Indeed, almost the totality of the synapses in the central nervous system use a chemical release of neurotransmitters which can individually take half a millisecond per connection, but varies depending on the type of synapses and other factors (Katz and Miledi, 1965).

The delay between the stimulus and its response is usually called reaction time, and measured in milliseconds (Deary and Der, 2005). In our case, since we do not record the time between a stimulus and an action, but between a stimulus and a written response, we chose to be using the term *response time* (RT) to avoid ambiguity, and we expect delays in the order of seconds.

We do not claim that neither measuring the reaction times nor the response times is the ideal portrayal of what is really occurring within the brain while processing sentences, nor that it is an ideal measure in and on itself, and in consequence over-interpreting the absolute values of these measures must be avoided. Still, they remain a very ecological tool as they do not require any dedicated recording device and can be used for data with high variability, unlike more precise timing measures like electroencephalography. They are often sufficient to demonstrate the impact of various factors on information processing (Fitts, 1966; Lachman et al., 1974; Thorpe et al., 1996; Bowyer et al., 2009, for examples).

Another critical factor to consider is that the reaction times can vary with the age of the participants in many tasks. As a consequence, considering the age as a potential factor should not be ignored when measuring reaction times, in particular with participants under 15 years old (Hale, 1990; Deary and Der, 2005). We should take similar considerations for RT measures.

In this experiment, we consider the response times between an interlocutor and the participant's utterances to be an indicator of the cognitive cost of processing the interlocutor's utterance. Data will only be collected with participants above 18 years old to avoid potential RT related biases.

## 3. Experiment

### 3.1. Materials and Methods

This experiment follows a strictly similar protocol as the one introduced in a preliminary experiment, with the addition of the condition of the violations of the fourth maxim of Manner, and with additional participants in all conditions (Jacquet et al., 2018).

#### 3.1.1. Participants

Eighty six native English-speakers familiar with textual conversations through messenger softwares (Skype, Telegram, Messenger or others) agreed to participate.

Most of them lived in North America (48), and in Europe (31). Three lived in Australia, two in Africa and two in Central America. They were recruited thanks to the help of contacts on the different continents. These contacts had to find one or more voluntary persons, of different gender whenever possible.

All participants were adults between 18 and 45 years old (*M* = 25, *SD* = 5.7). 46 of them were males, while 40 were females.

Participants had different backgrounds to avoid generating too much of a bias that could come from specialized professions or academic backgrounds. Results of our questionnaire on this question revealed very varied backgrounds in the general fields of Arts, Sciences, and Services. 24 did not answer this question.

#### 3.1.2. Variables

##### 3.1.2.1. Factor—maxim violations

The main discriminating factor between our conditions was the type of the Gricean maxim that was violated (the first maxim of Quantity, the maxim of Relation and the fourth maxim of Manner) by the experimenter (referred to as the actor) during the conversations with each participant. The conversation order (AI actor first or Human actor first), and the gender of the participants were also considered to be potential factors and were controlled.

##### 3.1.2.2. Main variable—response times

Our main dependent variable was the delay in seconds (the response time) between the moment the experimenter sent an utterance and the moment the participants sent their reply.

Since the length of the messages could potentially influence the response time, we designed a mathematical correction that was applied to all the recorded response times to remove the amount of delay that was likely caused by the number of characters in the sentences. We used a multiple linear regression model (with interaction) between the length of the experimenter's utterance and the length of the participant's reply on the observed delays. The model was inferred from the discussions between the participant and the human actor (which had no intentional violations of Grice's maxims) to create predictions of the delay as it should be without violations.

This model allowed us to calculate a theoretical delay (D) for each of the participants' responses.

(1) $$\begin{array}{r} D=\left(w\times C_{e}\right)+\left(x\times C_{p}\right)+\left(y\times C_{e}C_{p}\right)+z \end{array}$$

where *C*_*e*_ is the length of the experimenter's utterance, and *C*_*p*_ the length of the participant's utterance (both in number of characters). *w*, *x*, *y* and *z* are the coefficients of the model.

This theoretical delay was then removed from the observed delay. The resulting difference was then used to test our hypotheses and represented the deviation from normal (human-like) response times.

(2) $$\begin{array}{r} \Delta d=d-D \end{array}$$

where *d* is the observed delay.

##### 3.1.2.3. Secondary variable—identification percentage

The percentage of correct identification of the AI actor was also recorded to be compared to 50% (Random Chance). A value above random chance meant that the actor was perceived to be machine-like. A value around a random chance indicated that the actor was recognized to be human-like (Participants were unable to distinguish the two actors correctly).

##### 3.1.2.4. Control variables

Some other variables were recorded to control any potential bias. These control variables included the participants' gender, their age, the duration of each conversations (in seconds), the self-evaluated knowledge about AI (a Likert scale from 1 to 7) and self-evaluated knowledge about computer science (Likert scale from 1 to 7), and finally the confidence in their guess in the Turing Test (Likert scale from 1 to 7).

#### 3.1.3. Procedure

Since the experiment was entirely online, participants did not need more than a computer with an internet connection to be able to participate and could do so at home. To avoid adding bias to the recording of the response times, mobile devices could not be used to participate in this study, since typing speed can be influenced by the kind of device used. The chat where the experiment happened was hosted on a private French server and had been created for this study. Participants were required to communicate on Likert scales their knowledge in AI and Computer Science, their field of study or career, their gender and their age. This information was only sent to the server if consent was explicitly given for participating in the experiment.

Once in the *ChatBox*, the experimenter (displayed as “Moderator”) explained in detail the rules participants had to respect during the conversations as well as their task.

All conversations could only last up to 15 min, during which the participants could decide to stop the conversation if they had guessed which actor (either AI or human) they were talking to.

Both interlocutors (AI and human) were displayed with the same name (Andrew), and both tried to portray the same fictional character. This was done to invite the participants to ask questions to the fictional character instead of questioning the actors themselves. This meant that both actors would provide the same semantic information to the participant (since the character they're portraying is the same), but in different ways.

As we have mentioned before, there was actually no AI in our study. The two actors were, in fact, the same person (a male student in experimental psychology). The two roles only differed in their behavior regarding violations of the maxims. The experimenter in the human role had to behave like a “normal” human, without voluntarily adding violations. In the AI role, the experimenter was constrained in his behavior, and was not able to answer normally, but instead had to follow guidelines designed to produce as many violations of the required kind for the condition as possible, and in consequence to change the feeling of humanness given to the participant, since we expected this constrained behavior to feel closer to that of an AI. For each sentence, the actor indicated if a voluntary violation had been introduced because of these constraints.

The choice not to use an actual artificial intelligence during this test was motivated by the desire to restrict the differences between our conditions to the ones we could control. Vocabulary differences, syntax issues, or a defective understanding of the participant's utterances could have added further violations during the conversations that would have been difficult to predict in our protocol. It is also for the same reason we kept the same experimenter for the two roles.

As the Turing Test is about free-flowing conversations, it was impossible to keep them strictly identical between conditions, and even between participants within the same condition. To avoid strong biases, any new information given about the character of Andrew that had not been anticipated was written down so that the same information could be used again in different conversations. Participants sometimes reported being a bit surprised to see the same information between the two conversations.

Participants were able to discuss with both interlocutors (in random order), and each participant was assigned to one of the three conditions randomly (see the conditions below). The use of smileys, hypertexts, and double-posts was not allowed. Utterances (both from participants and actors) could not contain more than 255 characters.

#### 3.1.4. Conditions

A summary of the conditions and their expected effects is available in Table 1.

**Table 1:** Experiment summary.

	**Human**	**Machine**	**Hypothesis**
Quantity	No Violation	Lack of information	Small effect
Relation	No Violation	Generic utterances	Strong effect
Manner	No Violation	Random order of clauses	Intermediary effect

*Summary of the experiment along with the expected effects of the conditions on the response times and on the Turing test scores*.

##### 3.1.4.1. Quantity

The constrained behavior of the AI actor in this condition was the obligation to respond with too little information compared to what would be expected of a “normal” reply.

1. Participant: Anything planned for this evening?
2. Andrew: No idea. You? (alternative answer could have been: “Not really. What about you?”)

or

1. Participant: Do you have pets?
2. Andrew: Maybe. Do you? (alternative answer could have been: “I have two, yes. Do you?”)

This condition was expected to produce violations of the first maxim of Quantity^11^.

We expected this constraint would produce a small increase (if any at all) in the RT of the participants, and a low ratio of good identification in the Turing Test (close to a random chance).

##### 3.1.4.2. Relation

The constrained behavior of the AI actor in this condition was to avoid using contextual information from past messages. In cases where too little information is provided by the participant to generate a relevant reply, the machine actor could only reply with generic messages:

1. Participant: Do you like to read?
2. Andrew: I do yeah. Do you?
3. Participant: Yeah I do.
4. Andrew: Ok. What are you up to tonight? (Alternative answer could have been “What kind of books do you like?”)

or

1. Andrew: Do you have children?
2. Participant: Yeah I have two.
3. Andrew: Cool. Do you like games? (Alternative answer could have been “Cool. What are their names?”)

This condition was expected to produce violations of the maxim of Relation^12^.

We expected this constraint would produce a larger increase in the RT of the participants, and that it would increase the ratio of good identifications of the machine in the Turing Test (because of low humanness).

##### 3.1.4.3. Manner

The constrained behavior of the AI actor in this condition was to randomly swap the order of clauses within complex utterances containing at least two of them. Be it around connectors like *and, or*, commas, periods or any other delimiter of individual clauses.

1. Participant: What are you up to?
2. Andrew: Probably getting some sleep and then watching TV. Not much. (Alternative answer could have been: “Not much. Probably watching TV and then getting some sleep.”)

This condition was expected to produce violations of the third maxim of Manner (“Be orderly”).

We expected this constraint would produce a moderate increase in the RT of the participants, as well as an intermediate ratio of good identification in the Turing Test (between the two previous maxims).

### 3.2. Results

In total, 2,095 utterances written by the participants were recorded. One thousand and eighteen came from the discussions with the human actor, and 1,077 came from the discussions with the AI actor. Two male participants (Condition: Relation) were removed from the analysis as their conversation did not allow the AI actor to produce intentional violations matching their condition. One female participant (Condition: Relation) was removed for the same reason, and another female participant (Condition: Manner) asked to be removed from the study later. In conversations with the AI actor, 275 utterances were preceded by utterances with violations of the maxim of Quantity, 114 were preceded by utterances with violations of the maxim of Relation, 164 were preceded by utterances with violations of the maxim of Manner and the remaining 525 did not follow any violations.

Only the utterances coming from the discussions with the AI actor were used in the ANOVA (Type III).

Equal variances between groups were tested for the *post-hoc* analyses of the ANOVA using the Fisher test of equal variances. If groups had significantly different variances, comparison of means was analyzed with a Welch Two-Sample *t*-test, otherwise with a Two-Sample *t*-test.

Their *p*-values were adjusted using the Holm-Bonferroni correction to avoid type I errors (noted from now on *p*_*Holm*_).

We used Chi-Squared tests to compare the results of the Turing Test against a hypothetical distribution of 50% chance of correct identifications.

The Likert scales of self-evaluation on the knowledge in AI and in computer science were analyzed with the Kruskal-Wallis test by Ranks. The confidence scores were similarly analyzed.

The age of participants and the duration of the conversations were analyzed depending on the condition and gender with a Kurskal-Wallis test as well.

#### 3.2.1. Linear Model

The model generated using the conversations with the human actor resulted in the following equation (*R*^2^ =.4):

(3) $$\begin{array}{r} D=\left(0.15\times C_{e}\right)+\left(0.36\times C_{p}\right)-\left(0.0004\times C_{e}C_{p}\right)+9.2 \end{array}$$

by replacing in (1) *w* with 0.15, *x* with 0.36, *y* with 0.0004, and *z* with 9.2.

#### 3.2.2. Control Variables

There was no influence of the conditions on the confidence score reported by the participants ($\chi_{\left(2,N=86\right)}^{2}=0.04,p=0.98$), but there was a slight tendency for females to report lower scores of confidence than males ($\chi_{\left(1,N=86\right)}^{2}=3.19,p=0.07$).

Across the conditions, there was no significant difference in the distribution of the knowledge in AI ($\chi_{\left(1,N=86\right)}^{2}=1.4,p=0.5$) and in Computer Science ($\chi_{\left(1,N=86\right)}^{2}=0.3,p=0.86$), yet female participants reported significantly lower scores of Computer Science knowledge than male participants did ($\chi_{\left(1,N=88\right)}^{2}=8.3,p<0.01$).

The distribution of ages was not significantly different between the conditions ($\chi_{\left(2,N=86\right)}^{2}=2.2,p=0.32$) or between genders ($\chi_{\left(1,N=86\right)}^{2}=2.9,p=0.08$).

Finally, the duration of the conversations when participants were talking to the machine actor was not significantly different between conditions ($\chi_{\left(2,N=86\right)}^{2}=2.8,p=0.25$) nor was it different between genders ($\chi_{\left(1,N=86\right)}^{2}=0.35,p=0.55$).

#### 3.2.3. Response Times

We did not detect any significant double interaction between the type of violations, the order of the conversation and the gender on Δ*d* (*F*_3, 1062_ = 1.3, *p* = 0.26), but a significant interaction was found between the type of violations and the gender (*F*_3, 1070_ = 3.8, *p* < 0.01).

For the main effects, only the type of violation significantly influenced Δ*d* (*F*_3, 1070_ = 7.26, *p* < 0.001), but there was also a tendency for the gender to have an influence (*F*_1, 1070_ = 3.84, *p* = 0.07).

Because of the interaction between the gender of the participant and the type of violation, and considering the significant difference of self-evaluated knowledge in computer science between genders (that we noticed above), we also conducted another ANOVA to test the potential effect of the interaction between the type of violation and the self-evaluated knowledge in computer science (Low: ≤ 3, corresponding to below the first Quartile, and High: ≥5, corresponding to above the third Quartile). The influence of this interaction on Δ*d* was not significant (*F*_3, 634_ = 0.266, *p* = 0.85), nor could we find a main effect of the knowledge in computer science (*F*_1, 640_ = 1.14, *p* = 0.29).

A summary of the following analyses on the interaction between the type of violation and the gender of the participant is available in Table 2.

**Table 2:** Effect of Violations and Gender on Δ*d* (in seconds).

	**Mean (s)**	**SD (s)**	**SE (s)**	**Count**	***t***	***p*_*Holm*_**	
**NO VIOLATION**							
General	−1.20	17	0.75	525			
Males	−1.36	17	1.0	276			
Females	−1.02	18	1.1	249			
**QUANTITY**							
General	0.937	19	1.2	275	−1.54	0.12	
Males	3.11	21	1.8	143	−2.19	0.05	^*^
Females	−1.41	17	1.5	132	0.21	–	
**RELATION**							
General	7.57	24	2.2	114	−3.75	0.001	^***^
Males	3.88	16	2.1	62	−2.23	0.05	^*^
Females	12.0	30	4.1	52	−3.05	0.01	^**^
**MANNER**							
General	2.68	21	1.6	164	−2.16	0.06	
Males	5.48	23	2.6	78	−2.48	0.05	^*^
Females	0.15	19	2.0	86	−0.52	–	

*No violation refers to participant utterances in the conversation with the AI Actor following messages in which there had been no violation (Control). The t and p_Holm_ compare the means of each group to their respective control (For example, quantity males to no violation males)*.

* *p_Holm_ < 0.05*;

** *p_Holm_ < 0.01*;

*** *p_Holm_ < 0.001*.

##### 3.2.3.1. General

Δ*d* was significantly longer following utterances with a violation of the maxim of Relation than following utterances with no violation (*t*(140) = −3.75, *p*_*Holm*_ < 0.001), while we noticed a strong tendency for the a longer Δ*d* for violations of the maxim of Manner (*t*(237) = −2.16, *p*_*Holm*_ = 0.06), and no significant effect of the violations of the maxim of Quantity (*t*(504) = −1.54, *p*_*Holm*_ = 0.12).

##### 3.2.3.2. Interaction with the gender

**Figure 1:**
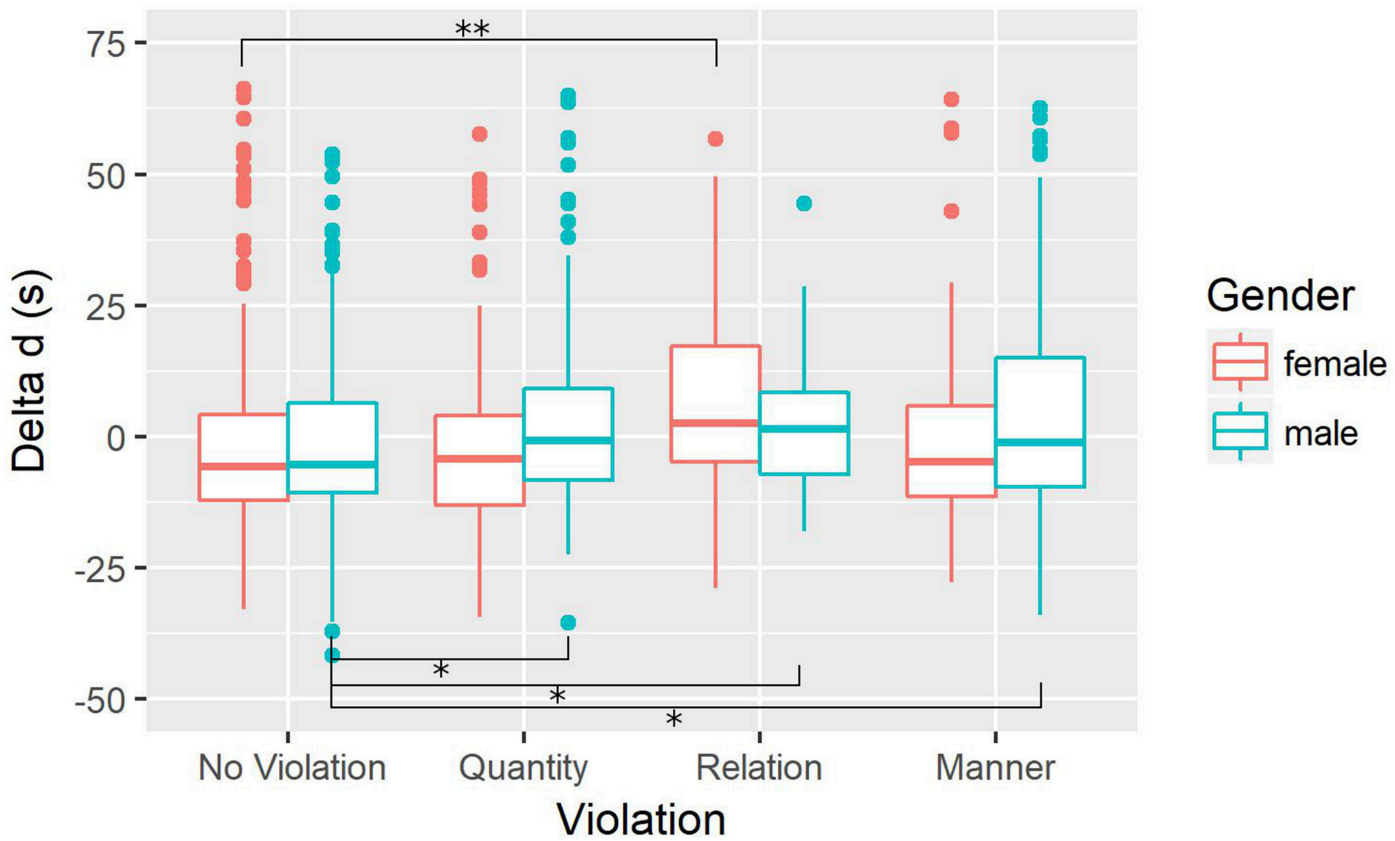
Effect of violations and gender on Δ*d* (in seconds). “No Violation” refers to participant utterances in the conversation with the AI actor following messages in which there had been no violation (Control). **p_*Holm*_ < 0.01, *p_*Holm*_ < 0.05.

The boxplot of this interaction is shown in Figure 1.

No significant difference was found depending on the gender after applying the Holm-Bonferroni correction, neither for violations of the maxim of Quantity (*t*(268) = −1.96, *p*_*Holm*_ = 0.20), nor for violations of the maxim of Relation (*t*(76) = 1.76, *p*_*Holm*_ = 0.25), nor for violations of the maxim Manner (*t*(162) = −1.64, *p*_*Holm*_ = 0.25).

##### 3.2.3.3. Utterances from female participants

Δ*d* was significantly longer following utterances with a violation of the maxim of Relation than following utterances with no violation (*t*(59) = −3.05, *p*_*Holm*_ = 0.01).

Other differences were not statistically significant.

##### 3.2.3.4. Utterances from male participants

For males, all conditions increased the RT compared to utterances without violations: for violations of the maxim of Quantity (*t*(236) = −2.19, *p*_*Holm*_ = 0.05), for violations of the maxim of Relation (*t*(336) = −2.23, *p*_*Holm*_ = 0.05), as well as for violations of the maxim of Manner (*t*(102) = −2.48, *p*_*Holm*_ < 0.05).

#### 3.2.4. Turing Test

In the following section, all percentages of correct identification are compared to a random chance (50%). A summary is shown in Table 3.

**Table 3:** Effect of violations and gender on the results of the Turing test.

	**Correct**	**Wrong**	**Percent**		**χ^2^**	**p**	
Total	55	31	64%		6.7	0.01	^**^
**QUANTITY**							
General	17	13	57%		0.53	0.47	
Males	7	9	44%		0.25	0.62	
Females	10	4	71%		2.6	0.10	
**RELATION**							
General	20	7	74%		6.3	0.01	^**^
Males	9	5	64%		1.6	0.20	
Females	11	2	85%		6.2	0.01	^**^
**MANNER**							
General	18	11	62%		1.7	0.19	
Males	12	4	75%		4.0	0.05	^*^
Females	6	7	46%		0.077	0.78	

*Correct refers to participants having successfully found the AI actor, while wrong refers to the remaining participants*.

* *p < 0.05*;

** *p < 0.01*;

^***^p < 0.001.

In general, participants managed to identify the AI actor easily (63%, χ^2^ = 6.7, *p* < 0.01). Yet, it varied depending on the condition and the gender of participants.

For the maxim of Quantity, there was no significant difference compared to a random chance for males (44%, χ^2^ = 0.25, *p* = 0.62), although there was a slight tendency for females (71%, χ^2^ = 2.57, *p* = 0.10).

For the maxim of Relation, there was no significant difference compared to a random chance for males (56%, χ^2^ = 1.6, *p* = 0.20), but there was a significant difference for females (86%, χ^2^ = 6.2, *p* < 0.01). In general, the difference compared to a random chance was also significant (74%, χ^2^ = 6.3, *p* < 0.01).

For the maxim of Manner, there was a significant difference compared to a random chance for males (75%, χ^2^ = 4, *p* < 0.05), but there was none for females (46%, χ^2^ = 0.1, *p* = 0.78).

## 4. Discussion

Generally speaking, it is worth noting that even though the ANOVA detected a significant interaction between the gender and the type of violation, the *post-hoc* analysis failed to highlight precisely in which condition gender differences contributed to this interaction. In consequence, gender differences considered below should be taken lightly, and only as hypotheses.

While we did not expect to find differences depending on gender, we included this factor in our protocol and in the discussion of the results below since an effect of the gender has previously been observed with social robots in Human-Machine Interaction experiments (Powers et al., 2005; Park et al., 2011; Tay et al., 2014) and was also present in our preliminary experiment (Jacquet et al., 2018). While the intensity of the effect depends on the study, gender differences when interacting with an artificial conversational partner seem to remain, especially since behavior seems to be different depending on the displayed gender of the artificial agent itself. In our case, the artificial agent was introduced as a male partner: Andrew.

In any case, if such differences between genders existed in our study, it was not caused by a potentially greater knowledge of computer science in males compared to that of females, as there was neither any interaction nor any main effect of this factor on the RT.

### 4.1. Quantity

The results of the experiment are for this condition partially coherent with our hypothesis stating that violations of the first maxim of Quantity would only trigger a small effect. Indeed, in general, there was only a slight increase (which was not significant), mainly driven by male participants (significant, while there was no significant difference for females).

It is worth noting that the correct identification percentage of males in this condition was actually worse than a random chance (44% success rate, compared to 71% for females in the same condition), meaning that males found the machine actor more human-like than the human actor, if only slightly (Although difference from a random chance was not significant).

These findings are coherent with the results reported by Saygin and Cicekli (2002). Indeed in their experiment, violations of the maxim did produce a feeling of humanness rather than an artificial feeling. In consequence, it is probably safe to assume that conversation partners are rather used to contributions that do not provide as much information as they expected, at least in conversations that are not task-oriented. Still, such violations gave participants the impression that their conversational partner was bored, upset or not very talkative, but not artificial.

Regarding male participants, their results are instead coherent with those of Engelhardt et al. (2006) which indicated that violations of this maxim could confuse the listener. Still, their results might not be directly translatable to those given by our experimental protocol as ours did not involve the need for the participants to act on the information given, but rather to guess on the general feeling of the humanness of the conversational partner.

If RT are indeed correlated to the cognitive cost required to infer the meaning of an utterance, it would mean that, in general, this cognitive cost, is very small indeed (Δ*d* = 0.937 s, *SD* = 19 s), as we predicted it would be. Yet, since males did not seem to perceive this increase as a sign of a lack of humanness, we can interpret that an increase in cognitive cost might not directly mean a decrease in the humanness of a conversational partner. This too might depend on the context in which this increase happened.

### 4.2. Relation

Female participants were much more sensitive to violations of the maxim of Relation than males, for they have shown the greatest increase in their RT in that condition. The recorded RT was also increased (*M* = 3.88 s, *SD* = 16 s), but to a lesser extent than with female participants (*M* = 12.0 s, *SD* = 30 s).

To our knowledge, a difference between genders in this context has not been discussed explicitly in the literature, neither to confirm nor to refute it. Yet, females show a tendency to ask more questions than male in conversations (Fishman, 1980), and they also tend to be brief compared to questions from males (Winter, 1993). This would explain why females might notice violations of the maxim of Relation more than males in our experiment, especially since it has been shown that girls are generally ahead of boys in language skills, which seems to be increasing with age (Eriksson et al., 2012). While the AI-actor could easily answer detailed questions without violating the maxim of Relation, he was incapable of answering short questions relying on the context of the conversation. Indeed, situations where such violations could arise required that the participants did not reference earlier messages or ideas without including in their utterances an indication of what they were talking about, like can be seen in this example^13^ (male participant):

1. Andrew: Anything planned for this evening?
2. Participant: Playing with my children for a few hours after I get home from work before bedtime and then watching Big Brother Canada with my wife.
3. Andrew: That sounds quite nice. How many children do you have?
4. Participant: 3
5. Andrew: Cool. What do you like to do in general?

In this example, the AI actor had no idea what the “3” in (4) is referring to anymore. Since he had no idea either of what they had been talking about before, he tried to give a generic answer to “3”: “Cool,” and kept moving the conversation forward with another generic question: “What do you like to do in general?” To this, the human actor would have instead replied “Cool, how old are they?” or “Cool, what are their names?”

While in the previous example the machine-actor was only once in a difficult situation, conversations with female participants could generate these situations much more often^14^:

1. Participant: I'm cooking.
2. Andrew: Oh cool, what are you making?
3. Participant: Can you guess?
4. Andrew: Well, it's not really an easy one.
5. Participant: Let us leave it to chance, what comes in mind?
6. Andrew: I'm not really sure to be honest.

In this example, the machine-actor finds itself in a difficult situation in both (4, 6). Indeed, since he cannot use the context of the conversation, he no longer knows what he has to guess in (3) and in (5). In this context for comparison, the human actor would have at least tried to give some food names.

The RT show that these violations had, in general, a strong effect, which is coherent with the idea that RT are correlated with the cognitive cost required, in our case, to switch topics or continue the conversation after decontextualized replies. The results in the Turing Test confirm what has been observed by Saygin and Cicekli (2002): Indeed, the great majority of participants considered the Actor producing these violations as less human (74% of correct identifications in total, 85% of successful identifications for women alone, and 64% for males, though the latter were not significantly different from a random chance).

Another measure of the cognitive load within utterances exists in the literature: surprisal^15^ (Hale, 2001; Levy, 2008). In these studies, surprisal is most often used at the level of the word, the surprisal being, in this case, the negative log probability of this word given its context, in particular its syntactic context. It is entirely possible that participants do not only predict the words they expect to see in a sentence but also, at a higher level, the replies they expect to receive within a conversation, given the context of the conversation itself, following the same principle: as the negative log probability of an utterance. This could explain why violations of the maxim of relation have the largest increase of RT, as the replies given by the actor in this condition are the farthest from what could have been expected of a conversational partner since it did not hesitate to switch topics. A similar pragmatic effect has been shown when studying the negation (Nordmeyer and Frank, 2015), showing that expected negations were not as difficult to process as unexpected ones. In this experiment, there was a strong correlation between RT and surprisal.

### 4.3. Manner

A significant effect has been found in the RT in this condition, but only for males who had their highest RT on average (*M* = 5.48 s, *SD* = 23 s), and the results in the Turing Test show a similar effect, as males managed to identify the AI actor significantly better than a random chance in this particular condition.

This was at least partially coherent with our hypothesis: this condition producing an intermediate effect between the previous two conditions. Yet, it seems that this condition had little effect on females in general despite the fact that all participants who succeeded in identifying the AI actor in this condition gave comments like “It's just that the flow felt weird and somewhat disconnected.”

It is possible that this was caused by a lack of actual violation in some cases of inversion^16^:

1. Andrew: Do you play that?
2. Participant: Used to, not anymore though
3. Andrew: What made you quit? Alright.

Indeed in (3), the difference between “What made you quit? Alright” and “Alright. What made you quit?” might not be very striking for the reader. In other cases, violations of the fourth maxim of Manner ended up making the participants misinterpret what the actor was saying^17^:

1. Participant: I'm doing well thank you, is there something you would like to talk about?
2. Andrew: What are you up to? I'm not really sure actually.
3. Participant: It's a bit of the same thing here, so. What is your current hobby?

In this case it is obvious to see that the flow of the conversation was disturbed by the swapping of Andrew's two clauses around the interrogation mark, and thus that a Violation of the maxim of Manner was produced, creating an ambiguity. Indeed, if we take (2) in its displayed sequential order, he moved on with the conversation before answering the participant's question.

It could feel like what Andrew was saying was that, first, he ignored the question asked by the participant “is there something you would like to talk about?” to ask directly “What are you up to?” The rest of his reply could mean that he was not sure what the participant might be up to, or that Andrew did not know what himself was going to be doing. The latter option seems to be the most likely considering the participant's answer, since they continued with (3), expressing that it was the “same thing here.” Yet, Andrew said nothing about what he was up to, since he only answered, in the wrong order, the participant's previous question about what he would like to talk about in (1).

Another interpretation could be that they did understand that he was answering their question in (1), and thus answered in turn that they did not know what to talk about either. Yet they immediately asked about Andrew's hobbies, ignoring his question about what they were up to.

Finally, yet another interpretation could be that they understood that Andrew meant “We could talk about what you are up to, but I'm not sure really,” which would also explain their reply.

Thus, in all three interpretations, there has been a misunderstanding, as the participant seems to have in one case imagined more information than Andrew actually provided, and in the other interpretations simply ignored Andrew's question.

Since all the information was available to the participant in such inversions, it is possible that they could form their interpretations of what Andrew meant fairly easily, despite this interpretation not necessarily be in line with what Andrew meant to say. This could partially explain why it did not produce such a strong effect for females. Their greater language skills (Eriksson et al., 2012) could have made it possible for them to generate an interpretation of the sentence more easily and with less processing costs than males, who might have remained a bit stuck on such utterances.

### 4.4. Linear Model

One could initially put in question the quality of the linear model in representing the participants typical response time, because of it's R squared (*R*^2^ = 0.4). Yet it is important to remember that at no point did we intend to make a perfect model of this process. The only goal of this model was to remove the effect of the length of sentences in our RT while keeping the variability of other factors. We are well aware that a number of different factors unrelated to our experimental protocol could play a part in the increase of the response times, including but not limited to memory recollection, or the time required in order to process the semantic content of the utterance, and other processes that cannot be easily predicted. All these things considered, our model was still an important device to use as the length of the sentences alone accounted for about 40% of the variability of the data. It is also worth noting that it did not contribute to the gender differences we observed. Indeed, predictions given by the model for utterances without violations in the conversations with the machine-actor were not significantly different between male and female participants.

### 4.5. Future Works

In this paper, we have only tested our hypothesis on the three maxim that seemed to have effects of different intensities on the feeling of humanness conveyed when they were violated. Other maxims could be studied, since some of them also contribute to an artificial feeling of the conversational partner when violated (Saygin and Cicekli, 2002), namely the second maxim of Quantity^18^. The second maxim of Manner^19^ could also be a good candidate for future studies as violating it would likely have a strong effect on the response times as well, but unlike the second maxim of Quantity, its violations have a strong positive effect on the humanness of the conversational partner.

We could also imagine testing other conversational expectations that are not directly Gricean, but share similar concepts of violations, like the expectations related to conversational politeness (Culpeper and Terkourafi, 2017) which have been shown to have an effect in human-machine interaction (Masson et al., 2017b).

Another technique, along with the recording of the response times, could involve the use of eye-trackers to investigate the fixations of the participants for each utterance (Groen and Noyes, 2013, for an example of this technique). It is likely that the participants would spend more time fixating surprising and obscure utterances. The main disadvantage of this technique is the fact that it requires having both the equipment and the participant physically present, which could make the general setting a lot less ecological compared to our protocol.

Regarding the potential differences observed depending on the gender of the participants, we believe another experiment involving fictive characters of different genders could be relevant in order to generate conditions with same-gender conversations and others with mixed-gender conversations as Mulac (1989) has shown that people of different genders do not necessarily behave in the same way depending on the gender of their conversation partners. In our case it was always a male character.

### 4.6. Conclusion

Our experiment seems to indicate that using the Turing Test along with recordings of the RT of participants is a relevant tool in studying online conversations. Indeed, using RT gives a much finer granularity to the collected data, bringing it from the level of the conversation (with the Turing Test) to the level of individual utterances (with the RT). It also contributes to investigating the idea that RT in a conversation might indeed be correlated to some extent to the cognitive cost of processing an utterance, and of generating a relevant reply.

We can also see that violations significantly increasing the RT of participants generally were associated with an increased machine-like effect in the identification from the Turing Test, with the only exception being violations of the first maxim of Quantity for males, adding further evidence that RT are a good tool to measure deviations from typical human interaction.

Unlike other evaluation methods, the ecological aspect of our protocol makes it stand out as participants are quite used to discussing in textual conversations with someone they do not actually see. Besides, they are given the opportunity to interact directly with the agent they are required to judge, instead of judging excerpts from previously recorded conversations, offering more variations of strategies, and contributing to the ecological aspect of the setting.

We believe that this experimental protocol constitutes a new way of evaluating conversational agents within online conversations, either compared to humans or compared to other artificial agents. Another benefit of using this method is that RT could potentially be analyzed while the conversation is happening, without having to wait for conversation samples to be analyzed by multiple human judges at a later date.

## Ethics Statement

All participants signed an online informed consent form before they were able to participate in the experiment. This consent form explained the general context of the experiment, that no implicit personal data (such as the IP) was collected, and that all conversations would remain private and would not be used beyond the scope of this study. Sharing the specific pieces of conversation shown in this paper has been approved by their specific authors. All participants were informed that they could opt-out of the experiment at any time and could ask us to remove their contribution. One participant so far has requested this. Participants were debriefed at the end of the experiment, including about the fact that they did not speak to an actual AI. The protocol of this experiment has been reviewed and approved by the ethical committee of the Laboratoire Cognitions Humaine et Artificielle (EA 4004-CHArt), Université Paris 8. Experimenters declare having respected the recommendations of the APA's ethical principles and code of conduct.

## Author Contributions

BJ designed the experiment, collected, and analyzed the data. BJ and JB wrote the draft of the manuscript and participated in the conceptual elaboration of the experiment. FJ offered his critiques and reviewed the manuscript.

### Conflict of Interest Statement

The authors declare that the research was conducted in the absence of any commercial or financial relationships that could be construed as a potential conflict of interest.

## Footnotes

^1^Woebot Labs Inc., https://woebot.io

^2^X2AI Inc. http://x2ai.com/

^3^Cleverbot is available at: https://www.cleverbot.com/

^4^Zo is a conversational agent developed by Microsoft. Conversing with it is possible through twitter: https://twitter.com/zochats

^5^Watson exists in the shape of a conversational agent API developed by IBM, allowing businesses to train their own version for specific purposes. https://www.ibm.com/watson/how-to-build-a-chatbot

^6^Images or GIFs that usually replace a facial expression, can be a reference to popular culture or many other types of contents. They are usually meant to convey humor.

^7^Personal assistant developed by Apple.

^8^Personal assistant developed by Microsoft.

^9^Artificial Intelligence developed by IBM.

^10^This conversation is from a public message on Twitter, available at https://twitter.com/zochats/status/1009141014827761664. It is possible to chat with the chatterbot directly on the same page, through private messages.

^11^“Make your contribution as informative as is required (for the current purposes of the exchange).”

^12^“Be relevant.”

^13^This participant accepted to have parts of his conversation made public following the experiment.

^14^This participant accepted to have parts of her conversation made public following the experiment.

^15^We would like to thank Reviewer 1 for suggesting a discussion on our results in the light of expectation-based models of language processing.

^16^This participant accepted to have parts of her conversation made public following the experiment.

^17^This participant accepted to have parts of her conversation made public following the experiment.

^18^“Do not make your contribution more informative than is required.”

^19^“Avoid ambiguity.”
